# Brown seaweed *Cystoseira* *schiffneri* as a promising source of sulfated fucans: Seasonal variability of structural, chemical, and antioxidant properties

**DOI:** 10.1002/fsn3.2130

**Published:** 2021-01-16

**Authors:** Abdelkarim Benslima, Sabrine Sellimi, Marwa Hamdi, Rim Nasri, Mourad Jridi, Didier Cot, Suming Li, Moncef Nasri, Nacim Zouari

**Affiliations:** ^1^ Laboratory of Enzyme Engineering and Microbiology National Engineering School of Sfax (ENIS) University of Sfax Sfax Tunisia; ^2^ Higher Institute of Applied Biology of Medenine (ISBAM) University of Gabes Medenine Tunisia; ^3^ Higher Institute of Biotechnology of Beja (ISBB) University of Jendouba Beja Tunisia; ^4^ Institut Européen des Membranes IEM‐UMR 5635 University of Montpellier ENSCM CNRS Montpellier France

**Keywords:** antioxidant activity, brown seaweed, *Cystoseiraschiffneri*, fucoidans, seasonal variation, structural characterization

## Abstract

A fucoidan, sulfated polysaccharide, was extracted from the brown seaweed *Cystoseira schiffneri* during 4 harvest periods (December, April, July, and September) and studied for its structural and chemical properties. The *Cystoseira schiffneri* fucoidan (CSF) showed important variation in sulfate content ranging from 7.8% in December to 34.8% in July. This was confirmed by Fourier transform infrared and nuclear magnetic resonance spectroscopies showing characteristic signals of sulfated polysaccharides. Molecular mass of the CSF varied as a function of season from 3,745 in December to 26,390 Da in July. Gas chromatography–mass spectroscopy showed that CSF fractions were “mannogalactofucans” composed mainly of mannose, fucose, and galactose with low levels of other monosaccharides. Moreover, interesting in vitro antioxidant activities that depend on the harvest season were noted for CSF. Thus, the present work might contribute to establish criteria for extracting bioactive fucoidans from an endemic Tunisian seaweed *C. schiffneri*.

## Graphical abstract

A novel fucoidan, mannogalactofucan, was extracted from the brown seaweed Cystoseira schiffneri. Mw of extracted fucoidans increased from 3,745 at December to 26,390 Da at July. The fucoidans showed interesting in vitro antioxidant activities that depend on the harvest season.

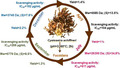

## 1 INTRODUCTION

Natural bioactive molecules from marine resources have shown many health beneficial effects. Hence, a growing interest was attributed to marine organisms (Chaula et al., 2019; Wijesekara et al., 2011), from which seaweeds were known for their functional polysaccharides (Sanjeewa et al., 2017; Sinurat & Rosmawaty, 2015; Zhao et al., 2018).

The sulfated fucans, also known as fucoidans, represented fucose‐containing sulfated polysaccharides that were widely extracted from brown seaweeds. They were reported to have many interesting bioactivities, such as antioxidant (Ashayerizadeh et al., 2020), anticancer (Thinh et al., 2018), anti‐inflammatory (Hadj Ammar et al., 2015), antiangiogenic (Liu et al., 2020), and antibacterial (Ashayerizadeh et al., 2020) activities. Fucoidans were nontoxic, biodegradable, and biocompatible compounds approved by the Food and Drug Administration (FDA). In fact, they were applied in many nutraceuticals and functional foods (Tanna & Mishra, 2019; Vo & Kim, 2013). Moreover, fucoidans were used as food additive for emulsion stabilization (Shi et al., 2020) and as fruits conservative (Duan et al., 2019). These polysaccharides were characterized by variable, irregular, and heterogeneous structures (He et al., 2020; Pradhan et al., 2020). In fact, the main chain could be homogenous, formed only by fucose monomers, or heterogeneous, composed of various monosaccharides and uronic acids. Monomers were mostly linked by bonds of type α(1 → 2). However, the bonds α(1 → 3) and α(1 → 4) were also reported. The fucoidans could be sulfated on C2, C3, and/or C4 (He et al., 2020), and they were generally linear but sometimes branched with a single or short chain of fucopyranose (Ale et al., 2011; Berteau & Mulloy, 2003; Chen et al., 2017). In addition, fucoidan structures were reported to be variable as a function of species, seasons, geographical location, climatic conditions, extraction methods, and age of seaweeds (Berteau & Mulloy, 2003; Li et al., 2017). These structural variances influenced biological activities of fucoidans, although the structure/activity relationship remains poorly understood.

Since the main source of fucoidans is the extracellular matrix of the Fucales (*Ochrophyta*, *Phaeophyceae*), the brown seaweed *Cystoseira schiffneri* Hamel was chosen for the first time as a matrix for their extraction. The Mediterranean endemic seaweed *C. schiffneri* is a taxon described in the islands of Djerba and Kerkennah from Tunisia, where it forms forests (Tsiamis et al., 2016). The species of the genus *Cystoseira* are perennial species with a monogenetic diplobiontic sexual cycle. The annual cycle of the Mediterranean *Cystoseira* passes through a growth period between February and May, a breeding period from June–July to August–September then a rest period between October and December (Lüning, 1993). Hence, the present work aimed to study the effect of annual cycle on the structural and chemical properties and antioxidant activities of *C. schiffneri* fucoidan (CSF).

## 2 MATERIAL AND METHODS

### 2.1 Reagents

1,1‐Diphenyl‐2‐picrylhydrazyl (DPPH•), 3‐(2‐pyridyl)‐5,6‐diphenyl‐1,2,4‐triazine‐disulfonic acid monosodium salt hydrate (ferrozine), butylated hydroxyanisole (BHA), ethylenediaminetetraacetic acid (EDTA), galacturonic acid, gelatin, FeCl_2_, BaCl_2_, NaCl, Na_2_CO_3_, trichloroacetic acid (TCA), H_2_SO_4_, HCl, H_3_BO_3_, 3,5 dimethylphenol, Tween‐80, 95% ethanol, absolute ethanol, methanol, chloroform, and acetone were purchased from Sigma Chemical Co. (St. Louis, MO, USA).

### 2.2 Sample collection and preparation

The samples of *Cystoseira schiffneri* Hamel were collected from Kerkennah Islands (Tunisia), more specifically around the point (34°39′30.07″N, 11°8′12.27″E) during low tide. Different samples were collected in December 2015, and April, July, and September 2016. The identity of the collected seaweed was validated by Pr. Asma HAMZA from the National Institute of Marine Science and Technology (Sfax, Tunisia). The seaweed fronds were washed thoroughly with seawater to eliminate sand, debris, and epiphytes and then transported to the laboratory in a dark plastic bag at a maximum of 12 hr. Once arrived, seaweed fronds were washed with distilled water to eliminate salts. Afterward, fronds were dried for 20 days in the dark at room temperature (25°C) until reaching stable moisture content before being ground using a coffee grinder (Moulinex, Mayenne, France) and sieved through a 0.2 mm mesh size. The seaweed powder was conserved for a maximum of 12 weeks in the dark and in a well‐sealed container at room temperature.

### 2.3 Fucoidan extraction

The CSF extraction was performed as previously described by Sellimi et al. (2014), with slight modifications. The *C. schiffneri* powder (50 g) was depigmented and defatted by maceration with 0.5 L acetone:methanol (7:3, v:v) (twice) and followed by 0.3 L chloroform (twice) for 24 hr at 30°C under constant stirring (200 rpm). Depigmented and defatted powders were air‐dried and then treated in 1 L 0.1 M HCl (pH = 3) for 2 hr at 60°C under constant stirring (250 rpm) for the CSF extraction. Next, the mixture was cooled at room temperature and centrifuged for 20 min at 4,000 × *g* at 4°C in a Rotofix 32 centrifuge (Hettich, Tuttlingen, Germany). The recuperated supernatant was mixed with 2 volumes of absolute ethanol and then left for 12 hr at 4°C to precipitate the fucoidan. Afterward, the fucoidan collected in the pellet by centrifugation (4,000 × *g*, 20 min, 4°C) was redissolved in distilled water, dialyzed using 14 kDa cutoff dialysis membrane from Sigma‐Aldrich (St. Louis, MO, USA), and finally lyophilized (Christ ALPHA 1–2 LD; Bioblock Scientific, Illkirch‐Cedex, France). The fucoidans extracted from *C. schiffneri* collected in December, April, July, and September were named FD, FA, FJ, and FS, respectively.

### 2.4 Chemical analyses

The total neutral sugar content was determined using the method of DuBois et al. (1956). To a solution of 0.1 g/ml CSF, 1 ml 5% phenol solution and 5 ml 12 N H_2_SO_4_ were added. The mixture was incubated at 30°C for 20 min, and then, the optical density was measured at 490 nm (T70 UV‐visible spectrometer; PG Instruments Ltd., Lutterworth, England) against a standard curve prepared using glucose.

The uronic acid content was determined using the method of Scott (1979). To 300 µl of a solution of 0.1 g/ml CSF, 5 ml of 12 N H_2_SO_4_ and 300 µl of a solution containing 20 g/L NaCl and 30 g/L H_3_BO_3_ were added. The mixture was incubated for 40 min at 70°C, then cooled to room temperature for 1 hr before adding 200 µl 3,5‐dimethylphenol. After 10 min at room temperature, the absorbance was measured at 400 and 450 nm against a standard curve of galacturonic acid. Uronic acids (%) were calculated using Equation 1.

(1) $$\text{Uronic acids (}\%)=\left(\Delta A\times V\times D\times C_{s}\times 0.91\times 100\right)/\left(\Delta A_{s}\times m\right)$$

where ΔA is the difference in absorbance; V is the total solution volume (mL); D is the sample dilution; Cs is the standard concentration; ΔAs is the difference in the absorbance of the standard (100 μg/ml); m is the mass of the test sample (mg); and 0.91 is the constant conversion factor of the experimental determination of monosaccharides to polysaccharides (Scott, 1979).

The sulfate group content was determined by the BaCl_2_–gelatin method as described by Dodgson (1961). BaCl_2_–gelatin solution was previously prepared by dissolving 2 g gelatin in 400 ml distilled water at 70°C. After 12 hr at 4°C, 2 g BaCl_2_ was added and the mixture was left at room temperature for 3 hr. Then, a volume of 0.2 ml of 2 mg/ml CSF solution was mixed with 3.8 ml 4% (w/v) TCA and 1 ml BaCl_2_–gelatine solution. After incubation for 15 min at room temperature, the absorbance was measured at 350 nm against a standard curve of K_2_SO_4_.

The total phenolic content was determined using a slightly modified method described by Cicco et al. (2009). A volume of 100 µl of 2 mg/ml CSF solution was mixed with 100 µl 2 N Folin–Ciocalteu's reagent and 800 µl 5% (w/v) Na_2_CO_3_ solution. The mixture was incubated at 40°C for 20 min, and then, absorbancies were measured at 760 nm against a standard curve of phloroglucinol.

### 2.5 Elementary analysis

The elementary analysis of CSF was performed using an energy dispersive X ray (EDX) analyzer (X‐Max ^N^ SDD EDX instrument, Oxford, UK). Oxford AZTEC software 2011 (Oxford Instruments, Abingdon, UK) was used to analyze results. Previous to analysis, the samples were metalized by coating with a thin gold layer (5 nm) using a Quorum SC7620 metalizer (Quorum Technologies, Brighton, UK) for 45 s at 12 mA under argon flux.

### 2.6 Monosaccharide composition

The monosaccharide composition of CSF was determined using the gas chromatography–mass spectroscopy (GC‐MS) method. The fucoidans were hydrolyzed using 2 M TCA at 100°C for 3 hr. The obtained neutral sugars were reduced using 0.5 mM NaBH_4_ and acetylation was done by Ac_2_O and pyridine. The resulting alditol acetate mixtures were diluted with chloroform prior to analysis.

The GC‐MS analysis was realized using (Hewlett Packard 5980A; CA, USA) gas chromatograph interfaced to a 5970B mass selective detector and equipped with Agilent 19091S‐433 capillary column (30 m × 0.25 mm × 0.32 mm). Helium flow rate was fixed at 1 ml/min, and temperature of injection was 250°C. Oven temperature started at 120°C for 10 min and then raise to 280°C by 5°C/min. Finally, the temperature was fixed at 280°C for 30 min. The mass spectrometer temperature was 250°C, and the ionization potential was 70 eV.

### 2.7 Molecular weight distribution

The weight‐average molecular weight (Mw), number‐average molecular weight (Mn), and polydispersity index (PI) of CSF were determined using high‐performance size‐exclusion chromatography (HPSEC) Waters Alliance model GPCV2000 (Waters, Milford, Massachusetts, USA) equipped with a multi‐angle laser light scattering (MALLS) detector from Wyatt (Wyatt technology, Santa Barbara, CA, USA). The PI, which represents the ratio Mw/Mn is a measure of the heterogeneity of a sample based on size. Before injection, the apparatus was calibrated with toluene and normalized with polyethylene oxide (72 kDa) in 0.1 M NaCl, and samples were filtered through a 0.45 µm pore size membrane (Merck, Darmstadt, Germany). A volume of 100 µl 3 mg/ml sample was injected in a (7.8 mm × 300 mm) column TSK‐G2000 SWXL (Tosoh Bioscience GmbH, Griesheim, Germany). The eluent was 0.1 M NaCl at a flow rate of 0.5 ml/min. The adopted specific refractive index increment (dn/dc) was 0.155. Data were collected from the refractive index detector (DRI) and MALLS, and evaluated with the ASTRA software version 4.72.03 (Wyatt Technology).

### 2.8 Spectral analyses

A Fourier transform infrared (FTIR) spectrophotometer (Perkin‐Elmer, Norwalk, CT, USA) equipped with attenuated total reflection (ATR) accessory containing a diamond/ZnSe crystal was used to study CSF infrared spectra. The FTIR spectra were obtained, using 30 scans and 4 cm^−1^ resolution, in the range of 4000–600 cm^−1^ at room temperature and at a scan speed of 0.6 mm/s.

^1^H‐NMR analysis was recorded at 25°C on a Bruker 400 spectrometer (Bruker Biospin AG, Fallanden, Switzerland). The results were analyzed using MestRe Nova 5.3.0 (Mestrelab Research S.L., Santiago de Compostela, Spain) software. The fucoidans were deuterium‐exchanged by lyophilization with D_2_O and then examined as 1% (w/v) solutions in D_2_O (99.96%) (Euriso‐Top, Paris, France).

### 2.9 Thermogravimetric analysis

The thermogravimetric analyses (TGA) were monitored using thermogravimetric analyzer Q500 instrument (TA Instruments, Newcastle, DE, USA). Nitrogen flow rate was fixed at 60 ml/min. Samples were heated from 20 to 1,000°C at a heating rate of 20°C/min, and their mass was constantly measured with an accuracy of 0.01 mg. Thermograms presenting the weight loss due to sample decomposition caused by temperature are obtained with Platinum™ Software (TA Instruments).

### 2.10 Antioxidant activities

#### 2.10.1 DPPH• radical scavenging activity

The DPPH• radical scavenging activity of CSF was determined following the method of Kirby and Schmidt (1997), with slight modifications. Briefly, 500 µl of the sample solution at different final concentrations (0.125–1.5 mg/ml) was mixed with 125 µl DPPH• solution (0.02% (w/v) in ethanol) and 375 µl absolute ethanol. The mixture was homogenized vigorously and then kept for 1 hr in the dark; the absorbance was recorded at 517 nm. BHA was used as a positive standard. Control (without sample) and blank (without DPPH•) were prepared and DPPH• reduction was calculated following Equation (2).

(2) $$\text{DPPH}∙\text{radical - scavenging activity (}\%)=\left(\left(A_{\text{control}}+A_{\text{blank}}‐A_{\text{sample}}\right)/A_{\text{control}}\right)\times 100$$

Results of DPPH• scavenging activity are shown by IC_50_ values (µg/mL) defined as the extract concentration needed to scavenge 50% of DPPH•. Lower IC_50_ values indicated higher DPPH• radical scavenging activity.

#### 2.10.2 (Fe^2+^) chelating activity

The CSF capacity to complex the ferric ion was tested by the method of Carter (1971). Briefly, 100 µl CSF solution (0.1–1 mg/ml) was mixed with 50 µl 2 mM FeCl_2_ and 450 µl distilled water. After 3 min of incubation at room temperature, 200 µl 5 mM ferrozine was added. The mixture was vigorously shaken and then incubated at room temperature for 10 min before reading absorbencies at 562 nm. The positive standard was EDTA. Blanks without ferrozine and a control without sample were prepared and chelating ability was calculated following Equation (3).

(3) $${\text{(Fe}}^{2+}\text{) chelating activity (}\%)=\left(\left(A_{\text{control}}+\;A_{\text{blank}}‐A_{\text{sample}}\right)/A_{\text{control}}\right)\times 100$$

Results of (Fe^2+^) chelating activity are shown by IC_50_ values (µg/ml) defined as the extract concentration needed to chelate 50% of Fe^2+^. Lower IC_50_ values indicated higher (Fe^2+^) chelating activity.

#### 2.10.3 Ferric ion (Fe^3+^) reducing antioxidant power (FRAP)

The FRAP of CSF was evaluated as described by Yildirim et al. (2001). Sample solution (500 µl) prepared at different concentrations ranging from 50 to 500 µg/ml was mixed with 1.25 ml 0.2 M sodium phosphate buffer (pH = 6.6) and 1.25 ml 1% (w/v) potassium ferrocyanide. After incubation for 30 min at 50°C, 1.25 ml 10% (w/v) TCA was added and the mixture was then centrifuged at 11,000 × *g* for 10 min (Gyrozen, Gimpo, South Korea). Afterward, a 1.25 ml aliquot of the supernatant from each sample mixture was mixed with 1.25 ml Milli‐Q water prepared by Milli‐Q^®^ Advantage A10 Water Purification System (Millipore Sigma, MS, USA) and 0.25 ml 0.1% (w/v) ferric chloride solution in a test tube. The absorbance of the resulting solutions was measured at 700 nm. Blanks without FeCl_3_ were prepared, and BHA was used as a positive standard. The FRAP is shown by the extract concentration (EC_0.5_) providing 0.5 of absorbance at 700 nm.

## 3 STATISTICAL ANALYSIS

Each experience was performed in triplicate, and results were expressed as mean ± standard deviation. Analysis of variance (ANOVA) with one factor was done to compare results using SPSS Windows™ (version 17; SPSS Inc., Chicago, IL, USA). Results are considered different at a level of *p < *.05.

## 4 RESULTS AND DISCUSSION

### 4.1 Extraction yield

Table 1 shows that the extraction yields of *C. schiffneri* fucoidans ranged from 1% to 2.2% (DM basis). Table 2 presents the extraction yields reported in the literature for some other brown seaweed species. The reported extraction yields varied dramatically from 1.3 to ~16%. The obtained results corresponded well with the range of the values previously reported in some other research papers. However, some other extraction yields remained higher than the measured values.

**TABLE 1:** Extraction yield (g/100 g DM) and chemical composition (g/100 g DM) of the CSF fractions from different seasons

Parameters	Season			
	FD	FA	FJ	FS
Yield	2.2 ± 0.03^a^	1.4 ± 0.04^b^	1 ± 0.03^c^	1.3 ± 0.05^b^
Moisture*	4.8 ± 0.1^d^	5.6 ± 0.2^b^	7.8 ± 0.3^a^	4.9 ± 0.2^c^
Total sugars	73.9 ± 2.4^a^	69 ± 2.2^b^	52.8 ± 1.1^c^	74.9 ± 1.4^a^
Uronic acids	8.3 ± 0.04^a^	6.3 ± 0.2^b^	8.3 ± 0.2^a^	8.1 ± 0.2^a^
Total phenolics^†^	0.4 ± 0.04^d^	1 ± 0.02^a^	0.7 ± 0.02^b^	0.6 ± 0.1^c^
Ash	11 ± 0.4^c^	15.8 ± 0.6^b^	36.5 ± 1^a^	33.3 ± 1.5^a^
C	35.6 ± 3^b^	41.7 ± 2.1^a^	37.6 ± 1.3^b^	42.4 ± 3.5a
O	38.3 ± 1.1^b^	42 ± 1.8^b^	41.4 ± 1.4^b^	46.5 ± 1.4^a^
Na	7.5 ± 0.1^a^	1.4 ± 0.144^b^	0.7 ± 0.2^c^	1.5 ± 0.3^b^
S	4.5 ± 0.1^c^	7.8 ± 0.3^b^	10.4 ± 0.2^a^	4.6 ± 0.7^c^
*N*	0	0	0	0
Mg	0.7 ± 0.1^b^	2.2 ± 0.04^a^	0.3 ± 0.1^d^	0.6 ± 0.03^c^
K	0.4 ± 0.1^c^	0.5 ± 0.1^b^	1.3 ± 0.1^a^	0.3 ± 0.03^d^
Cl	8.2 ± 0.4^a^	0.8 ± 0.3^c^	4.2 ± 0.9^b^	0.8 ± 0.5^c^
Ca	4.8 ± 0.2^a^	3.6 ± 0.1^c^	4.1 ± 0.2^b^	3.5 ± 0.6^c^

Note

FD, FA, FJ, and FS represent the extracted fucoidans from *C. schiffneri* collected in December, April, July, and September, respectively.

^a,b,c,d^Different letters within different seasons of harvest (same column) indicate significant differences (*p* < .05).

* the moisture is expressed as g/100 g lyophilized fucoidan.

^†^ Total phenolics were expressed as g eq. phloroglucinol/100 g DM; each value represents the mean ± *SD* (*n* = 3).

**TABLE 2:** Literature data of some brown seaweed fucoidans

Parameters	Species	Value	References
Extraction yield (%)	*Saccharina longicruris*	1.3	Rioux et al., (2007)
	*Ascophyllum nodosum*	2.2	Rioux et al., (2007)
	*Padina* sp	2.1	Lim et al., (2014)
	*Cystoseira barbata*	5.5	Sellimi et al., (2014)
	*Turbinaria conoides*	8.8	Chattopadhyay et al., (2010)
	*Laminaria japonica*	~16	Zhao et al., (2018)
Total sugars (%)	*C. barbata*	50.8	Qu et al., (2019)
Uronic acids (%)	*L. japonica*	6.8	Chen et al., (2017)
	*L. japonica*	20.3	Zhao et al., (2018)
	*C. barbata*	7.1	Sellimi et al., (2014)
	*Undaria pinnatifida*	4.1	Koh et al., (2019)
Total phenolics (%)	*C. compressa*	1.4	Hentati et al., (2018)
Sulfate content (%)	*Fucus serratus*	34	Fletcher et al., (2017)
	*F. vesiculosus*	19	Fletcher et al., (2017)
	*A. nodosum*	15	Fletcher et al., (2017)
	*S. sculpera* (July)	1.6	Qu et al., (2019)
	*S. sculpera* (Mars)	0.4	Qu et al., (2019)
	*C. barbata*	22.5	Qu et al., (2019)
	*C. compressa*	14.7	Hentati et al., (2018)
	*U. pinnatifida*	22.8	Koh et al., (2019)
	*S. japonica (sterile)*	17	Vishchuk et al., (2012)
	*S. japonica (fertile)*	14	Vishchuk et al., (2012)
	*Alaria* sp. *(sterile)*	24	Vishchuk et al., (2012)
	*Alaria* sp. *(fertile)*	29	Vishchuk et al., (2012)
Mannose (%)	*C. barbata*	2.9	Sellimi et al., (2014)
	*S. japonica (sterile)*	47	Vishchuk et al., (2012)
	*S. japonica (fertile)*	5.8	Vishchuk et al., (2012)
	*Alaria* sp. *(sterile)*	8	Vishchuk et al., (2012)
	*Alaria* sp. *(fertile)*	1.2	Vishchuk et al., (2012)
Fucose (%)	*C. barbata*	44.6	Sellimi et al., (2014)
	*C. compressa*	62.4	Hentati et al., (2018)
	*U. pinnatifida*	~30	Koh et al., (2019)
	*S. japonica (sterile)*	32	Vishchuk et al., (2012)
	*S. japonica (fertile)*	63	Vishchuk et al., (2012)
	*Alaria* sp. *(sterile)*	54	Vishchuk et al., (2012)
	*Alaria* sp. *(fertile)*	48	Vishchuk et al., (2012)
Galactose (%)	*C. barbata*	34.3	Sellimi et al., (2014)
	*C. compressa*	24.2	Hentati et al., (2018)
	*U. pinnatifida*	~23	Koh et al., (2019)
	*S. japonica (sterile)*	9	Vishchuk et al., (2012)
	*S. japonica (fertile)*	22	Vishchuk et al., (2012)
	*Alaria* sp. *(sterile)*	38	Vishchuk et al., (2012)
	*Alaria* sp. *(fertile)*	50.4	Vishchuk et al., (2012)
Glucose (%)	*C. barbata*	7.6	Sellimi et al., (2014)
	*C. compressa*	7.7	Hentati et al., (2018)
	*U. pinnatifida*	~3	Koh et al., (2019)
	*S. japonica (fertile)*	7	Vishchuk et al., (2012)
Xylose (%)	*C. barbata*	4.2	Sellimi et al., (2014)
	*C. compressa*	4.5	Hentati et al., (2018)
	*U. pinnatifida*	~30	Koh et al., 2(019)
	*S. japonica (sterile)*	8	Vishchuk et al., (2012)
	*S. japonica (fertile)*	2.2	Vishchuk et al., (2012)
Rhamnose (%)	*S. japonica (sterile)*	4	Vishchuk et al., (2012)
Mw (kDa)	*L. japonica*	6.5	Zheng et al., (2018)
	*F. serratus*	1,336–2,024	Fletcher et al., (2017)
	*F. vesiculosus*	1,184–1,789	Fletcher et al., (2017)
	*A.nodosum*	1,274–1,469	Fletcher et al., (2017)
DPPH• scavenging activity (CI50, µg/mL)	*C. barbata*	~650	Sellimi et al., (2014)
	*Sargassum polycystum*	759	Palanisamy et al., (2017)
	*U. pinnatifida*	200	Mak et al., (2013)
FRAP (CE_0.5_, µg/mL)	*C. barbata*	400	Sellimi et al., (2014)
	*L. japonica*	>1,100	Qu et al., (2014)
	*Lessonia nigrescens*	>1,100	Qu et al., (2014)
	*L. trabeculata*	>1,100	Qu et al., (2014)
	*A. mackaii*	~400	Qu et al., (2014)
	*Ecklonia maxima*	~500	Qu et al., (2014)
(Fe2+) chelating activity (CI50, µg/mL)	*L. japonica*	>3,500	Wang et al., (2009)
	*C. barbata*	~300	Sellimi et al., (2014)

It is worthy to note that the fucoidan content varied considerably according to season. The highest extraction yield was measured for FD, while the lowest one was obtained for FJ, which suggests that the best period to extract the highest CSF content would be winter. Similarly, Fletcher et al. (2017) reported that December is the best harvest month in terms of the highest fucoidan yield. In contrast, Men’shova et al. (2012) reported for *P*. *pavonica* that fucoidan extraction yield was more important in July.

The highest fucoidan extraction yield measured in December seems to be related to abiotic factors, such as decrease in temperature, illumination, and salinity. According to Skriptsova (2015), abiotic factors, such as water temperature, mineral concentration, salinity, and illumination, had an influence on the fucoidan accumulation. It was also reported that *C. schiffneri* showed seasonal variation for pigment and lipid contents that were likely to be related to abiotic factors (Salem et al., 2017). On the other hand, the variation of fucoidan content between seasons may be attributed to the seaweed growth cycle. It was reported that fucoidans played a crucial role in gamete extrusion and that were released immediately before spores, which might be the main cause of their decrease in July (Skriptsova, 2015).

### 4.2 Chemical analysis

The moisture, total sugar, uronic acid, total phenolic, and ash contents were presented in Table 1. The CSF were mainly composed of neutral sugars ranging from 52.8% to 74.9%, while uronic acids showed comparable levels with respect to the harvest season. Ash content varied considerably depending on the season (11%–36.5%), while markedly low contents of phenolic compounds were measured. Literature data for some other brown seaweed species showed higher neutral sugar content and a wide range of uronic acid content from 4.1% to 20.3%. Similarly, low levels of phenolic compounds were also reported (Table 2).

The elementary composition of CSF was also shown in Table 1. High levels of carbon and oxygen were measured, while nitrogen was not detected that indicated the absence of proteins in the extracted CSF. Fletcher et al. (2017) also noted negligible nitrogen levels for the extracted fucoidans from *Fucus serratus*, *F. vesiculosus,* and *A*. *nodosum*. The sulfur content that ranged between 4.5% and 10.4% suggested that CSF was a sulfated polysaccharide. The obtained results showed that C:S:O ratios were 1:0.12:1.07 for FD, 1:0.19:1.01 for FA, 1:0.28:1.10 for FJ, and 1:0.11:1.10 for FS. The relatively higher oxygen content as compared to carbon content would be due mainly to sulfate groups containing 4 oxygen atoms (SO_4_^2–^) and also to uronic acids containing one more oxygen atom (C_6_H_9_O_7_) as compared to neutral sugars (C_6_H_9_O_6_). Fletcher et al. (2017) reported lower C:S ratios for *F. serratus* (0.2–0.31), *F. vesiculosus* (0.23–0.42), and *A. nodosum* (0.25–0.6) as compared to the CSF fractions. Table 1 shows that Na, Mg, Cl, K, and Ca were present despite the dialysis of CSF fractions, which suggested their high chelating ability (Fletcher et al., 2017).

**FIGURE 1:**
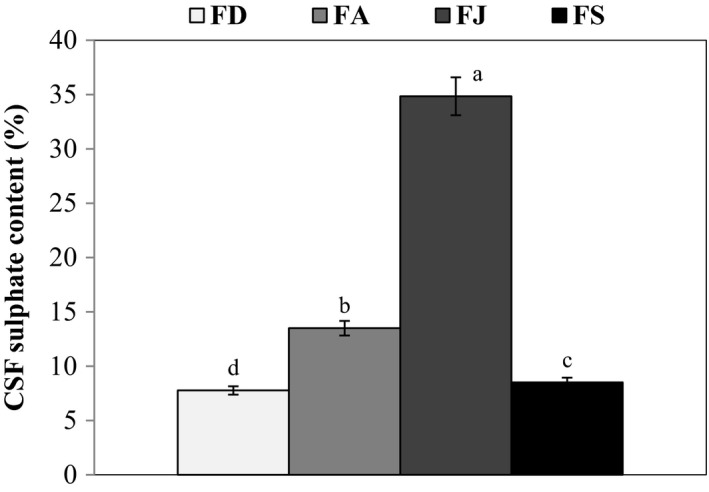
Sulfate content of the extracted fucoidans from *C. schiffneri* (CSF) collected in December (FD), April (FA), July (FJ), and September (FS)

Furthermore, the sulfate content in CSF fractions showed a fluctuation between seasons (Figure 1). The highest sulfate content was measured for FJ, while the lowest one was obtained for FD. Similar findings were reported in the literature (Table 2), where the sulfate content varied remarkably as function of harvest season and therefore as function of reproductive cycle. According to Honya et al. (1999) and Skriptsova et al. (2010), the sulfate content of fucoidan was higher during the reproductive stage similarly to the obtained results. However, as it was mentioned in Table 2 for some other brown seaweed species, the highest sulfate content was obtained in sterile stage. Hence, sulfate content of fucoidans seems to be species‐specific. It is interesting to note that the fucoidan sulfate content is a crucial characteristic that affects their biological activities. In fact, Haroun‐Bouhedja et al. (2000) reported that sulfate content less than 20% lead to a complete loss of antiproliferative and anticoagulant activities of fucoidan. The obtained results suggested that the summer months were the best period for CSF extraction, in terms of the highest sulfate content.

### 4.3 Monosaccharide composition

Table 3 shows monosaccharide composition of CSF fractions, which presented high levels of mannose, fucose, and galactose. Low contents of xylose, arabinose, and mannitol were measured only for FA and/or FJ. In comparison with previous studies, different results were obtained, where fucoidans were composed mainly of fucose and galactose with lower or no amounts of mannose, glucose, xylose, and rhamnose (Table 2). On the other hand, homogenous fucoidan formed only by fucose monomers was also isolated for *F*. *distichus* (Bilan et al., 2004). According to Ale et al. (2011), brown seaweeds contained very complex fucans structures named galactofucans, which had comparable amounts of fucose and galactose. These galactofucans consisted mainly of galactose and mannose units with a terminal end of glucose or xylose well as branch points made up of fucoses and uronic acids. Besides, other structures were also highlighted for brown seaweeds, such as *Himanthalia lorea* and *A*. *nodosum* fucans composed only of fucose, xylose, and uronic acids (Ale et al., 2011). To our knowledge, this is the first report of a fucoidan containing mannose as the main monomer. Hence, the isolated fucoidan could be named “mannogalactofucan.” However, a survey of the literature showed that the monosaccharide composition of fucoidan isolated from *S. japonica* was more complicated at sterile stage, while no difference was shown between the fucoidans of *Alaria sp* between the sterile and fertile stages (Table 2). The obtained results showed that CSF composition was more complex at fertile stage (April and July); thus, the monosaccharide composition profile seemed to be species‐specific.

**TABLE 3:** Monosaccharide composition (%) of the CSF fractions from different seasons

	Season			
	FD	FA	FJ	FS
Mannose	47.6	38	34.8	36.5
Fucose	25.2	23.8	23.7	28
Galactose	22.7	25.1	23	29.4
Glucose	4.5	5.4	13.5	6.1
Xylose		3.6	2.9	
Arabinose		3.2	2.1	
Mannitol		0.9		

Note

FD, FA, FJ, and FS represent the extracted fucoidans from *C. schiffneri* collected in December, April, July, and September, respectively.

### 4.4 Molecular weight distribution

Mw, Mn, and PI of CSF were determined, and results are shown in Table 4. FJ presented the highest Mw value, while FD had the lowest one. The isolated fucoidans were polydisperse (PI > 1), and FJ presented the highest PI. Comparable results were reported for fucoidans extracted from *L. japonica*. However, Mw of CSF remained very low as compared to other brown seaweed species (Table 2). Fletcher et al. (2017) reported that Mw fluctuation seems to be depending mainly on species rather than the harvest season. The low Mw for CSF seems to be interesting for bioactive effects, since fucoidans with low Mw showed many therapeutic potentials (Ale et al., 2011).

**TABLE 4:** Weight‐average molecular weight (Mw), number‐average molecular weight (Mn), and polydispersity index (PI) of the CSF fractions from different seasons

	Season			
	FD	FA	FJ	FS
Mw (Da)	3,745	6,585	26,390	6,779
Mn	744.6	3,932	1930	2,406
PI	5	1.7	13.7	2.8

Note

FD, FA, FJ, and FS represent the extracted fucoidans from *C. schiffneri* collected in December, April, July, and September, respectively.

### 4.5 Spectral analyses

**FIGURE 2:**
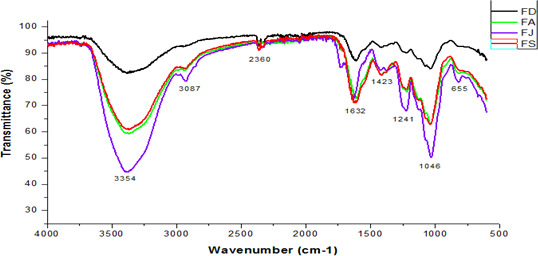
ATR‐FTIR spectroscopy of the extracted fucoidans from *C. schiffneri* collected in December (FD), April (FA), July (FJ), and September (FS)

The FTIR‐ATR spectroscopy analysis was performed to determine the specific CSF absorption bands. The CSF spectra showed characteristic bands of fucoidans at 655, 1,046, 1,241, 1,423, 1,632, 2,360, 3,067, and 3,354 cm^−1^ (Figure 2). The peaks detected at 3,354 and 3,067 cm^−1^ were assigned to O‐H and to C‐H stretching vibration bands, respectively. The signal at 1,046 cm^−1^ corresponded to C‐O‐C stretch vibration of glycosidic linkage. Moreover, the wavenumbers at 1,423 and 1632 cm^−1^ were assigned to C=O stretching vibration of uronic acids residues. Furthermore, the FTIR‐ATR profiles confirmed the presence of sulfate groups on the CSF fractions. Indeed, the absorption band at ~655 cm^−1^ suggested the presence of a C‐O‐S stretching band of axial sulfate groups and the signal, at 1,241 cm^−1^, suggested the presence of S = O stretching vibration of sulfate esters (Song et al., 2018).

**FIGURE 3:**
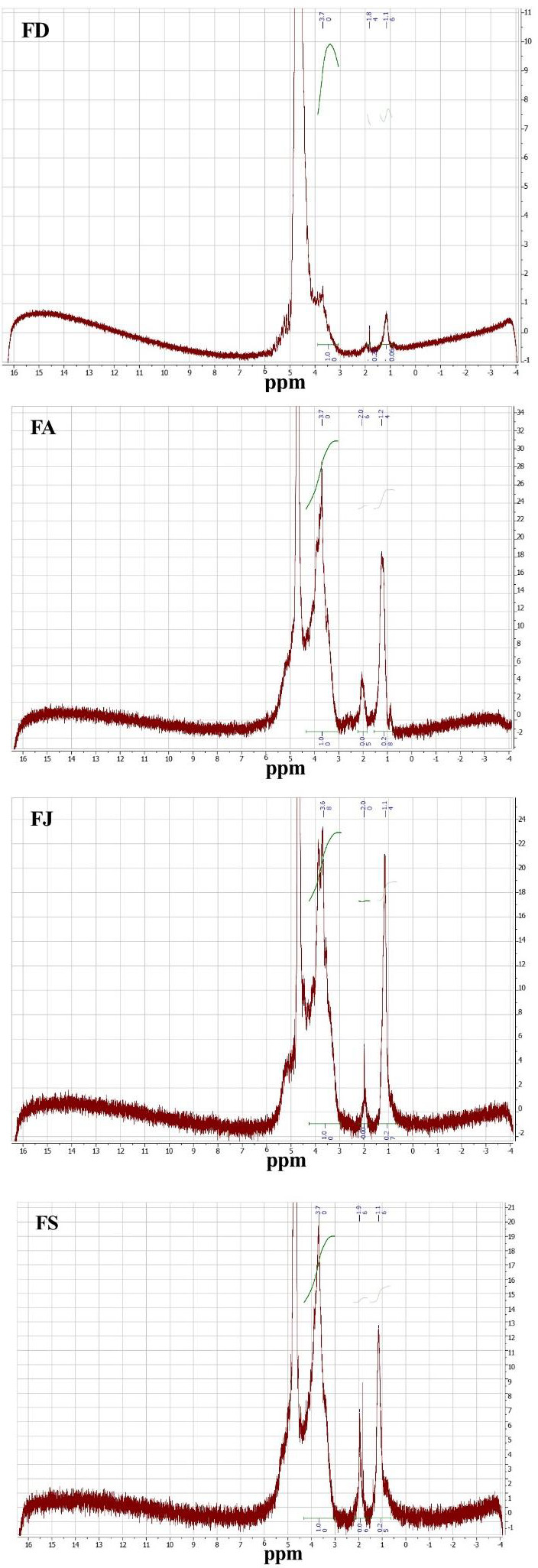
^1^h‐NMR spectra of the extracted fucoidans from *C. schiffneri* collected in December (FD), April (FA), July (FJ), and September (FS)

The ^1^H‐NMR spectroscopy was also used to determine the CSF configuration. The NMR spectra presented in Figure 3 showed characteristic signals of sulfated fucans for all analyzed CSF fractions, which was in concordance with the FTIR‐ATR results. The signals obtained at 5.64–5.03 ppm were assigned to H1 of fucopyranose and to C‐H protons of O‐substituted carbons (Synytsya et al., 2010). Ring protons (H2–H5) showed characteristic resonances between 3 and 4.5 ppm. Besides, the signals obtained at 1.4 and 1.2 ppm were attributed to methyl protons H6 of l‐fucopyranose (Synytsya et al., 2010). Kariya et al. (2004) reported that signals observed at 1.24 ppm were ascribed to fucosyl residues linked in (1–3). Furthermore, the signal obtained at 4.3 ppm could be attributed to the H4 of 4‐O‐sulfated residues (Kariya et al., 2004; Pereira et al., 1999) and the signals observed at 2.14–2.21 ppm could be ascribed to CH3 protons of O‐acetyl groups (Synytsya et al., 2010).

### 4.6 Thermogravimetric analysis

**FIGURE 4:**
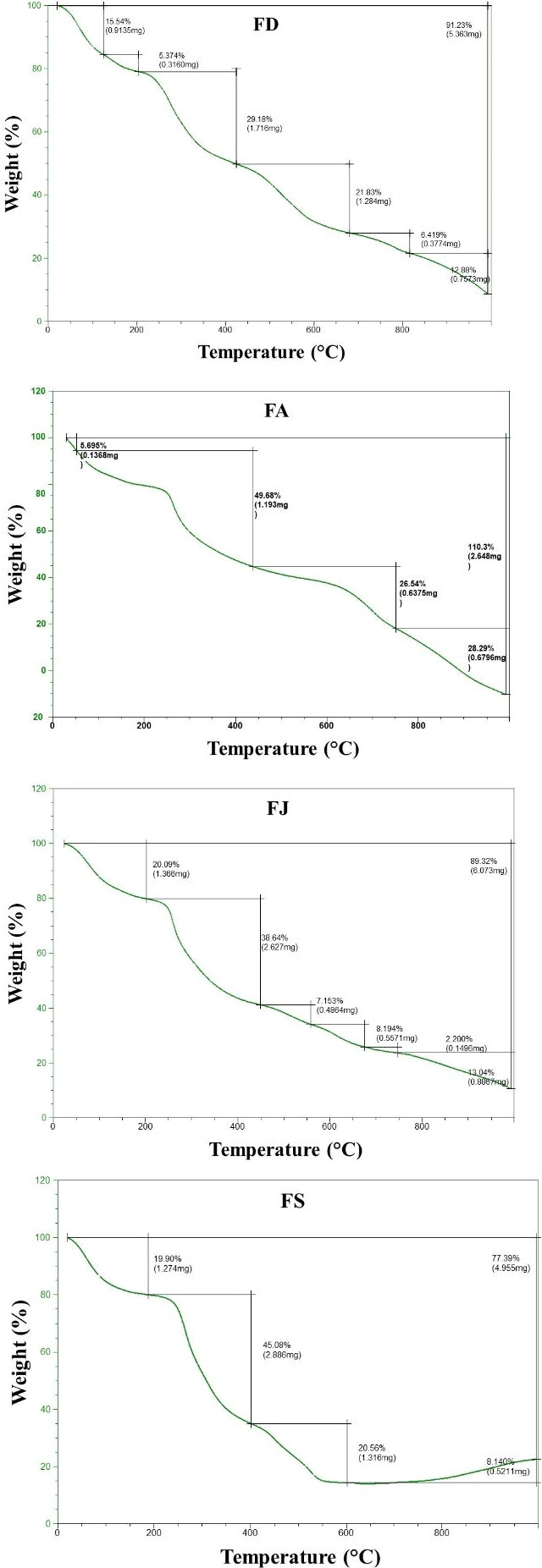
Thermogravimetric analysis of the extracted fucoidans from *C. schiffneri* collected in December (FD), April (FA), July (FJ), and September (FS)

The mass losses of CSF as a function of temperature were presented in Figure 4. According to Idris et al. (2012), three phases of degradation namely drying, pyrolysis, and char combustion were obtained. All the CSF fractions showed almost the same degradation profile. The first mass loss, due to humidity removal, was detected at temperatures ranging from 50 to 200°C (Mallick et al., 2018). The main CSF degradation (29.18%–49.68%) that was characteristic of the pyrolysis phase took place in the range of 400–450°C and which was assigned to the glycosidic monomers degradation. Depending on the harvest season of *C. schiffneri,* a significant difference in the CSF degradation temperature was noted, which may be due to their Mw or to the mineral level (Mallick et al., 2018). In fact, a positive correlation between Tmax and Mw was obtained (*R* ^2^ = 0.48). However, Mw seems to be not the only factor affecting the monomers degradation. According to López‐González et al. (2014), alkali metals act as catalyzers of combustion process. Finally, the Char combustion step started at temperatures above 500°C and it was characterized by small and multiple mass losses.

### 4.7 Antioxidant potential

Antioxidant activities of the CSF fractions were measured using three complementary tests: (i) DPPH• radical scavenging activity; (ii) (Fe^2+^) chelating activity, and (iii) ferric ion (Fe^3+^) reducing antioxidant power, which showed variation in a concentration‐depending manner (data not shown). Table 5 shows the IC_50_ and EC_0.5_ values of the measured activities. The FA was the most active fraction in the DPPH radical‐scavenging and (Fe^2+^) chelating tests, while FD and FS were the most active fractions in the FRAP analysis (Table 5). Comparable results for antioxidant activities were reported for other species of brown algae (Table 2). According to Mak et al. (2013), DPPH• radical scavenging activity was influenced by the Mw of polysaccharide. Additionally, monosaccharide composition, glycosidic linkage type, and sulfate group positions affected the radical scavenging activity (Li et al., 2008; Skriptsova et al., 2010). The (Fe^2+^) chelating activity seems to be related to the fucoidan structure. Van Acker et al. (2013) reported that (Fe^2+^) chelating activity of molecules depends on the presence of some functional groups (O‐H, C = O, ‐S‐, ‐O‐, COOH), as well as to their orientation. In fact, the group (SO_4_) in ortho position could chelate ferric ions. According to Raza et al. (2011), the polysaccharide hydroxyl groups were responsible for their reducing ability. Moreover, Qu et al. (2014) reported that FRAP of fucoidans was related to their sulfate content and Mw.

**TABLE 5:** Antioxidant activities of the CSF fractions from different seasons

	Season				Standards	
	FD	FA	FJ	FS	BHA	EDTA
DPPH• scavenging activity	192 ± 3^c^	104 ± 5^d^	611 ± 14^a^	266 ± 10^b^	14 ± 0.2^b^	
(Fe^2+^) chelating activity	>1,000	96 ± 3^c^	144 ± 6^b^	209 ± 12^a^		10 ± 0.2^e^
FRAP	71 ± 3^c^	304 ± 6^a^	147 ± 5^b^	63 ± 3^c^	25 ± 0.1^c^	

Note

FD, FA, FJ, and FS represent the extracted fucoidans from *C. schiffneri* collected in December, April, July, and September, respectively; results of DPPH• scavenging and metal (Fe^2+^) chelating assays are shown as IC_50_ values (µg/mL), defined as the extract concentration needed to scavenge 50% of DPPH• and to chelate 50% of Fe^2+^, respectively. The ferric ion (Fe^3+^) reducing antioxidant power (FRAP) is shown as the extract concentration (EC_0.5_, µg/mL) providing 0.5 absorbance at 700 nm; each value represents the mean ± *SD* (*n* = 3).

^a,b,c,d,e^Different letters within different seasons of harvest (same row) indicate significant differences (*p* < .05).

Overall, the CSF fractions showed interesting antioxidant potential with different action mechanisms in dependence to the harvest season. The Mw, sulfate group type, and monosaccharide distribution could be considered to be the main factors that influenced the CSF antioxidant activities. This leads to the need to match the algae harvest season with the desired characteristics of their fucoidans.

## 5 CONCLUSIONS

In the present study, a novel fucoidan named mannogalactofucan was extracted from the brown seaweed, *C. schiffneri* collected from Kerkennah Islands (Tunisia). The Mw, sulfate content, and monosaccharide composition of fucoidans varied considerably as function of the reproduction cycle. The variability in these parameters seems to affect the antioxidant activities. The present study suggested that the best time of *C. schiffneri* harvest could be in July to obtain fucoidans with the highest sulfate content, while December was the best harvest month allowing low Mw and high extraction yield of fucoidan. Thus, further investigations are necessary to better understand the structure–activity relationship and to investigate other potential activities.

## CONFLICT OF INTEREST

The authors declare that they have no conflict of interest.

## Data Availability

All authors confirm that the data supporting the findings of this study are available within the article.
